# Supplementary material for: Globus pallidus dynamics reveal covert strategies for behavioral inhibition
Source: eLife. 2020 Jun 10;9:e57215. doi: 10.7554/eLife.57215 (PMC7314538; doi:10.7554/eLife.57215)
Supplement: Transparent reporting form [file elife-57215-transrepform.docx]

***eLife’s* transparent reporting form**

We encourage authors to provide detailed information *within their submission* to facilitate the interpretation and replication of experiments. Authors can upload supporting documentation to indicate the use of appropriate reporting guidelines for health-related research (see [EQUATOR Network](http://www.equator-network.org/%20)), life science research (see the [BioSharing Information Resource](https://biosharing.org/)), or the [ARRIVE guidelines](http://www.plosbiology.org/article/info:doi/10.1371/journal.pbio.1000412) for reporting work involving animal research. Where applicable, authors should refer to any relevant reporting standards documents in this form.

If you have any questions, please consult our Journal Policies and/or contact us: [editorial@elifesciences.org](mailto:editorial@elifesciences.org).

**Sample-size estimation**

- You should state whether an appropriate sample size was computed when the study was being designed
- You should state the statistical method of sample size computation and any required assumptions
- If no explicit power analysis was used, you should describe how you decided what sample (replicate) size (number) to use

Please outline where this information can be found within the submission (e.g., sections or figure legends), or explain why this information doesn’t apply to your submission:

- No explicit power analyses were performed.

- There are no generally-accepted guidelines specifying how many neurons are appropriate for robust neural population analysis. One study found that tens of neurons were sufficient to identify the dominant population activity patterns (Williamson et al., 2016). Other studies have argued that the number of neurons should be determined by the neural task complexity, and found that reducing the number of neurons from 109 to 36 did not change the measured dimensionality (Gao and Ganguli 2015, Gao et al., 2017). Given this prior literature, the number of cells used in the present study (376) is likely to be fully sufficient for the analyses performed.

- The number of trials recorded in each session was the maximum number that rats were willing to perform the task.

References:

Gao P, Ganguli S. On simplicity and complexity in the brave new world of large-scale neuroscience. Curr Opin Neurobiol. 2015 Jun;32:148-55.

Gao P, Trautmann E, Yu B, Santhanam G, Ryu S, Shenoy K, Ganguli S. A theory of multineuronal dimensionality, dynamics and measurement bioRxiv 214262; doi: https://doi.org/10.1101/214262

Williamson RC, Cowley BR, Litwin-Kumar A, Doiron B, Kohn A, Smith MA, Yu BM. Scaling Properties of Dimensionality Reduction for Neural Populations and Network Models. PLoS Comput Biol. 2016 Dec 7;12(12):e1005141.

**Replicates**

- You should report how often each experiment was performed
- You should include a definition of biological versus technical replication
- The data obtained should be provided and sufficient information should be provided to indicate the number of independent biological and/or technical replicates
- If you encountered any outliers, you should describe how these were handled
- Criteria for exclusion/inclusion of data should be clearly stated
- High-throughput sequence data should be uploaded before submission, with a private link for reviewers provided (these are available from both GEO and ArrayExpress)

Please outline where this information can be found within the submission (e.g., sections or figure legends), or explain why this information doesn’t apply to your submission:

- We include a complete description of the number of experiments and the exclusion/inclusion criteria:

in the Results:

*Here we report behavioral and neural results for 6 rats for which we were able to obtain high-quality GP recordings as rats engaged proactive control. We selected for further analysis those behavioral sessions (n=63) with a significant proactive inhibition effect (i.e. longer RT when a Stop cue might occur; one-tail Wilcoxon rank sum test, p<0.05) and distinct GP single units (n=376 neurons included)*

and in the Methods:

*To avoid duplicate neurons, we did not include data from the same tetrode across multiple sessions unless the tetrode had been moved by > 100μm between those sessions. Based on waveform and firing properties we further excluded an additional 25 units that appeared to be duplicates even though the tetrode had been moved.*

**Statistical reporting**

- Statistical analysis methods should be described and justified
- Raw data should be presented in figures whenever informative to do so (typically when N per group is less than 10)
- For each experiment, you should identify the statistical tests used, exact values of N, definitions of center, methods of multiple test correction, and dispersion and precision measures (e.g., mean, median, SD, SEM, confidence intervals; and, for the major substantive results, a measure of effect size (e.g., Pearson's r, Cohen's d)
- Report exact p-values wherever possible alongside the summary statistics and 95% confidence intervals. These should be reported for all key questions and not only when the p-value is less than 0.05.

Please outline where this information can be found within the submission (e.g., sections or figure legends), or explain why this information doesn’t apply to your submission:

- Statistical tests and p-values are reported for every analysis, in Results and in figure legends.

- For behavioral data (Fig 1 and Fig 1 legend), we used non-parametric Wilcoxon signed rank tests to compare two related samples.

- For individual neurons, we used non-parametric Wilcoxon signed rank tests (for Fig.2 D,E,G) and binomial tests (Fig. 2B,F) (details are described in the Fig2 legend)

- For neural population analyses (Fig 3,4,5), we used non-parametric permutation tests, shuffling the trial conditions 10000 times for each test.

(For large datasets, or papers with a very large number of statistical tests, you may upload a single table file with tests, Ns, etc., with reference to sections in the manuscript.)

**Group allocation**

- Indicate how samples were allocated into experimental groups (in the case of clinical studies, please specify allocation to treatment method); if randomization was used, please also state if restricted randomization was applied
- Indicate if masking was used during group allocation, data collection and/or data analysis

Please outline where this information can be found within the submission (e.g., sections or figure legends), or explain why this information doesn’t apply to your submission:

- Samples were not allocated into experimental groups.

- Key analyses compare data between different trial types, which were randomly assigned by computer software on each trial.

**Additional data files (“source data”)**

- We encourage you to upload relevant additional data files, such as numerical data that are represented as a graph in a figure, or as a summary table
- Where provided, these should be in the most useful format, and they can be uploaded as “Source data” files linked to a main figure or table
- Include model definition files including the full list of parameters used
- Include code used for data analysis (e.g., R, MatLab)
- Avoid stating that data files are “available upon request”

Please indicate the figures or tables for which source data files have been provided:

We have uploaded data files and metadata to the public website *Figshare: https://figshare.com/articles/Globus_pallidus_dynamics_reveal_covert_strategies_for_behavioral_inhibition/12367541*
